# Measures of Adherence and Challenges in Using Glucometer Data in Youth with Type 1 Diabetes: Rethinking the Value of Self-Report

**DOI:** 10.1155/2017/1075428

**Published:** 2017-12-21

**Authors:** Karishma A. Datye, Niral J. Patel, Sarah S. Jaser

**Affiliations:** Department of Pediatrics, Vanderbilt University Medical Center, Nashville, TN, USA

## Abstract

**Purpose:**

The current study compares the relative strength of associations of different adherence measures with glycemic control in adolescents with type 1 diabetes, while highlighting the challenges in using more objective measures (i.e., glucometer data).

**Methods:**

Adolescents with type 1 diabetes (*n* = 149) and their caregivers completed a questionnaire measure assessing adolescents' adherence (Self-Care Inventory (SCI)) to the diabetes regimen. Adolescents' glucometers were downloaded to determine average blood glucose checks per day, as an objective measure of adherence. A measure of glycemic control (hemoglobin A1c (HbA1c)) was obtained as part of adolescents' regular clinic visits.

**Results:**

Adolescents' self-reported adherence to the treatment regimen was more strongly correlated with HbA1c than caregivers' reports of adherence. In multivariate analyses, both adolescents' self-report of adherence and average blood glucose checks per day (obtained via a glucometer) were significant predictors of HbA1c. Challenges to obtaining glucometer data were identified.

**Conclusions:**

The findings highlight adolescents' self-report of adherence using the SCI as a brief and meaningful measure to understand and improve adolescents' glycemic control, particularly when glucometer data is difficult to obtain.

## 1. Introduction

To achieve optimal glycemic control, the treatment regimen recommended for patients with type 1 diabetes (T1DM) is complicated and demanding, including multiple blood glucose checks per day, paying careful attention to carbohydrate intake and activity levels, and administering several doses of insulin per day [1]. It is critical for providers to understand their patients' adherence to therapy, given the positive association between adherence and glycemic control [2]. However, problems with adherence during adolescence are common, occurring in up to 93% of teens with T1DM [3]. For many adolescents, every blood glucose check is a test that they could fail, and many report feeling guilty about “bad” numbers [4] or avoiding diabetes care in front of peers [5].

Understanding patients' adherence to therapy allows providers to understand what challenges their patients are facing on a daily basis and to inform decisions about changes to insulin dosing. This becomes complicated in youth with diabetes, as multiple measures of adherence are available (e.g., caregivers' report, youths' report, glucometer or pump download, and log book), and the complexity of the treatment regimen makes it difficult for providers to evaluate adherence. Additionally, while some measures of adherence, such as glucometer or insulin pump data, provide seemingly objective data (i.e., number of blood glucose checks per day and number of boluses) [6, 7], these data can be challenging to obtain and alone do not provide information about associated barriers to specific aspects of the treatment regimen [8]. Previous studies have reported mixed findings as to the best measures of adherence: in one study of adolescents aged 12–17, self-reported adherence was most strongly linked with glycemic control [9], whereas another study with a sample of younger adolescents aged 10–14 found that parents' reports of adherence were the best predictor of glycemic control [10]. The study by Kichler et al. had a relatively small sample size (*n* = 76) and did not include parents' reports of adherence, and a large percentage of participants did not have complete glucometer data (27.6%) [9]. The study by Berg et al. included a larger sample (*n* = 252), with both mothers' and fathers' reports of adherence, but they also reported a considerable percentage of missing or unusable glucometer data (13%) [10]. Recent findings also demonstrate that frequency of blood glucose testing and insulin administration may increase in some youth prior to clinic visits [11]. This finding, known as “white coat adherence,” further complicates a provider's ability to assess adherence to therapy. Thus, the purpose of the current study was to build on previous findings by comparing the relative value of different measures of adherence in adolescents with T1DM and highlighting challenges related to obtaining complete glucometer data.

## 2. Methods

Data from two studies in which participants were recruited from the same pediatric diabetes clinic were combined for this secondary analysis. In study 1 (baseline data from a behavioral intervention study (*n* = 120)), participants were aged 13–17 and had been diagnosed with T1DM > 6 months (only one participant (from study 1) had been diagnosed for less than 12 months, and this participant had an HbA1c > 8%, suggesting that the honeymoon period was over), and in study 2 (a descriptive, cross-sectional study (*n* = 29)), participants were aged 11–21 and had been diagnosed with T1DM > 1 year (only data from participants aged 13–17 were included in the current analyses). Participants were excluded if they had other major medical or psychiatric conditions or if they were participating in other intervention studies. Study 1 enrollment criteria included hemoglobin A1c (HbA1c) between 8.0 and 12.0% (64–108 mmol/mol); study 2 had no HbA1c inclusion criteria. All participants were recruited following the protocol approved by the Institutional Review Board, and participants received compensation (gift cards) for completing surveys. The rate of participation was 65% in study 1 and 75% in study 2.

A combined total of 149 participants were enrolled into this study during routine T1DM clinic visits. In both studies, participants and their caregivers completed the Self-Care Inventory (SCI, 12), and caregivers provided demographic data. The SCI is a 14-item measure that assesses key elements of adherence to the treatment regimen for T1DM. Higher scores indicate greater adherence to the treatment regimen. In the current study, Cronbach's alpha was 0.71 for the adolescents' report and 0.73 for the caregivers' report.

A 30-day glucometer download was also obtained during participants' clinic appointments to calculate average blood glucose (BG) checks per day (if the meter was not available, participants estimated BG checks per day and this was documented separately). A 30-day download was chosen instead of a 14-day download given previous studies which showed that adherence to blood glucose monitoring improves as the clinic visit date approaches [11]. Glycemic control was measured using the adolescent's point-of-care HbA1c (DCA Vantage Analyzer manufactured by Siemens).

Attempts were made to obtain glucometer data from all participants at the time of enrollment, but many adolescents had incomplete glucometer data. The most common reason for incomplete data was that participants did not bring all of their glucometers to the visit (*n* = 42, 28.2%). Three participants brought no glucometer (2%), and one participant had a glucometer with only one reading. Seven (4.7%) did not bring their primary meter, and therefore, self-report data for the previous 7 days was obtained. Given concerns about accuracy of self-report, participants who had missing meters and those who did not bring their primary meters were excluded from analyses. The remaining 31 participants brought their primary glucometer to the clinic visit, but they had additional glucometers with readings at school or at a secondary caregiver's residence. Of the adolescents who brought all of their glucometers, we experienced problems with data downloads (*n* = 5), inaccurate dates in the glucometers (*n* = 5), and lost/broken glucometers within the past month (*n* = 3). Complete and accurate glucometer data (at least 28 days of usable data) were available for 94 participants (63.1% of the total sample). However, for data analyses, we included participants if they brought at least one meter with usable data (*n* = 138).

Bivariate analyses were conducted to determine the correlation between each measure of adherence (self-report, caregivers' report, and BG monitoring) and glycemic control. In addition, a series of *z*-tests were conducted to compare the relative strength of associations between indicators of adherence and HbA1c. Finally, a hierarchical linear regression analysis was conducted, predicting HbA1c from all three indicators of adherence, after adjusting for adolescents' age, sex, and treatment type (pump or injections). IBM SPSS version 23 was used for all analyses.

## 3. Results

Data from 149 adolescents and their caregivers were obtained (see Table 1 for demographic and clinical characteristics). There were no significant differences in adolescents' age, sex, or race/ethnicity between the two study samples. Further, the samples were not significantly different in the percent of participants using insulin injections versus pumps or the average checks per day. The only significant difference between study samples was in HbA1c (study 1 mean Hba1c = 9.2%, study 2 mean HbA1c = 8.6%). The mean HbA1c for adolescents from both studies was above the target range (HbA1c < 7.5%). In addition, we compared adolescents who brought all meters to the clinic visit to those who did not and found no significant differences in age, sex, race/ethnicity, treatment type (pump versus injections), or HbA1c.

As seen in Table 2, adolescents' self-report of adherence on the SCI was significantly related to caregivers' report of adherence and to average BG checks per day. Caregivers' reports of adherence were not significantly related to average checks per day. All three measures of adherence were significantly associated with glycemic control, with higher adherence related to lower HbA1c. Comparisons of the different measures revealed that adolescents' SCI had a significantly stronger association with HbA1c than caregivers' SCI (*z* = −2.31, *P* = 0.021). However, the association of HbA1c with average checks per day was not significantly stronger than that with adolescents' SCI (*z* = −1.31, *P* = 0.095) or caregivers' SCI (*z* = −1.30, *P* = 0.194).

Finally, the regression model predicting HbA1c was significant (*F*(129,6) = 6.47, *P* < 0.001), explaining 23% of the variance in glycemic control. As seen in Table 3, in the final model adjusted for youths' sex, treatment type, and age, average checks per day and self-reported adherence (SCI) were significant predictors of HbA1c. However, caregivers' report of adolescents' adherence was not significant in the multivariate analysis.

## 4. Discussion

In the current study of adolescents with T1DM, we examined different indicators of adherence in relation to glycemic control (HbA1c). In addition, we highlighted some of the challenges inherent in obtaining more objective measures of adherence, such as glucometer data. Although all measures were significantly related to HbA1c, in the multivariate model, self-report of adherence (SCI) and average blood glucose checks per day emerged as most strongly correlated with glycemic control.

These findings are in line with those of previous research by Kichler and colleagues [9], suggesting that the SCI is strongly correlated with glycemic control and offers another tool to assess adherence to therapy. Additionally, the SCI may be more informative than glucometer data, as it offers information on specific aspects of the treatment regimen (not only blood glucose monitoring). On the other hand, our findings differ from those reported by Berg and colleagues [10], in which parents' report of adherence was most strongly linked with glycemic control. This may be due to differences in the glycemic control of the samples; the range of HbA1c levels was much wider (4.9–13.9%) in the study by Berg et al. as compared to our study (6.2–11.5%), and this could reflect differences in adherence behaviors. Self-report is often overlooked as a metric of adherence given concerns about retrospective accuracy and reliability; however, our findings indicate that the SCI is a valuable tool for providers to assess and understand patients' adherence to therapy, and it may be more accurate than relying on caregivers' reports for older adolescents.

### 4.1. Clinical Implications

Using the SCI as a screening tool completed by patients prior to clinic visits may result in more productive conversations between providers and patients, as it gives providers an opportunity to address potential barriers to adherence. This information could be included in the medical record as part of patient-reported outcomes [12]. Further, it is important for providers to keep in mind that, although certain measures of adherence are viewed as more objective (glucometer or pump downloads), they may present problems with missing/inaccurate data. Adolescents often have more than one glucometer (e.g., one at school and one at the home of a secondary caregiver), which they may forget to bring to clinic visits, providing an incomplete picture of adherence. Additionally, youth may increase frequency of blood glucose testing prior to a clinic visit (white coat adherence) which may misrepresent their adherence to therapy [11]. Insulin pump downloads (BOLUS scores) may provide more accurate and meaningful data than glucometers [7], but using these excludes the considerable number of adolescents who do not use an insulin pump. Even with advances in diabetes management platforms for combining data from different devices (e.g., Glooko, SweetSpot, and TidePool), these require uploads from devices, and the current study highlights the difficulties in obtaining glucometer data that present challenges for providers to interpret and use these data.

Limitations of this study include the small sample size, cross-sectional study design, incomplete glucometer and pump data, and differences in eligibility requirements for the two studies that provided data. In addition, the reliability of the SCI in our sample was somewhat lower than that in other studies, but still within the acceptable range. Despite these limitations, the findings highlight the SCI as a brief and meaningful measure to assess adherence in adolescents with T1DM in order to improve glycemic control. Future research is needed to continue to assess measures of adherence for youth with T1D, including those using continuous glucose monitors and insulin pumps, to determine which measures are most predictive at different developmental stages.

## Figures and Tables

**Table 1:** Demographics and baseline clinical characteristics (*n* = 149).

Adolescents' baseline variables	Range	Mean (SD)
Age (years)	13–17	14.95 (1.40)
HbA1c (%)	6.2–11.5	9.04 (0.98)
Duration of diabetes (years)	0–16	6.07 (3.82)
*Demographic variables*		*N (%)*
Adolescents' sex		
Female		79 (53.0)
Adolescents' race/ethnicity		
White, non-Hispanic		126 (87.5)
Other		18 (12.5)
Treatment type		
Insulin pump		70 (47.0)
Injection		79 (53.0)
Annual household income (USD)		
Less than $39,999		41 (27.7)
$40–79,999		50 (33.8)
More than $80,000		57 (38.54)
Parental marital status		
Nonmarried/partnered		13 (20.0)
Married/partnered		52 (80.0)
Relationship to child		
Mother		121 (81.2)
Father		23 (15.4)
Other		5 (3.4)

**Table 2:** Descriptive statistics and correlations among glycemic control and measures of adolescents' adherence.

	1	2	3	4	5	6
(1) Adolescents' age (*M* = 14.95 (1.40))	—					
(2) Duration of diabetes (*M* = 6.07 (3.82))	0.02	—				
(3) HbA1c (*M* = 9.04 (0.98))	0.09	0.02	—			
(4) A-SCI (*M* = 3.61 (0.73))	−0.11	−0.03	−0.40^∗∗∗^	—		
(5) P-SCI (*M* = 3.59 (0.74))	−0.18^∗^	0.00	−0.23^∗∗^	0.44^∗∗∗^	—	
(6) BGM (*M* = 3.33 (1.85))	−0.21^∗^	−0.05	−0.29^∗∗∗^	0.25^∗∗^	0.17^∗^	—

HbA1c = hemoglobin A1c; A-SCI = adolescents' report of self-care inventory; P-SCI = parents' report of self-care inventory; BGM = blood glucose monitoring (average checks per day). ^∗^*P* < 0.05. ^∗∗^*P* < 0.01. ^∗∗∗^*P* < 0.001.

**Table 3:** Hierarchical linear regression predicting adolescents' HbA1c from measures of adherence.

Predictor	*β*	*R* ^2^	Δ*R*^2^
Step 1		0.03	0.03
Adolescents' sex	−0.13		
Treatment type	0.05		
Adolescents' age	0.07		
Step 2		0.22^∗∗∗^	0.20^∗∗∗^
Adolescents' sex	−0.15		
Treatment type	0.03		
Adolescents' age	−0.03		
BGM	−0.18^∗^		
Adolescents' SCI	−0.33^∗∗∗^		
Caregivers' SCI	−0.08		

*Note.* BGM = blood glucose monitoring (average checks per day); SCI = self-care inventory. The model value is adjusted *R*^2^. *β* = standardized beta. ^∗^*P* < 0.05. ^∗∗∗^*P* < 0.001.
